# Urinary Biomarkers as a Proxy for Congenital Central Hypoventilation Syndrome Patient Follow-Up

**DOI:** 10.3390/antiox11050929

**Published:** 2022-05-09

**Authors:** Marta Peruzzi, Matteo Ramazzotti, Roberta Damiano, Marzia Vasarri, Giancarlo la Marca, Cinzia Arzilli, Raffaele Piumelli, Niccolò Nassi, Donatella Degl'Innocenti

**Affiliations:** 1 Sleep Breathing Disorders and SIDS Centre, A. Meyer Children’s Hospital, 50134 Florence, Italy; marta.peruzzi@meyer.it (M.P.); cinzia.arzilli@meyer.it (C.A.); raffaele.piumelli@meyer.it (R.P.); niccolo.nassi@meyer.it (N.N.); 2 Department of Experimental and Clinical Biomedical Sciences “Mario Serio”, University of Florence, 50134 Florence, Italy; matteo.ramazzotti@unifi.it (M.R.); roberta.damiano@unifi.it (R.D.); marzia.vasarri@unifi.it (M.V.); giancarlo.lamarca@meyer.it (G.l.M.); 3 Newborn Screening, Biochemistry and Pharmacology Laboratory, A. Meyer Children’s Hospital, 50134 Florence, Italy

**Keywords:** CCHS, rare disease, oxidative stress, urinary biomarkers

## Abstract

Congenital Central Hypoventilation Syndrome (CCHS) is a rare genetic disorder of the autonomic nervous system and in particular of the respiratory control during sleep. No drug therapy is, to date, available; therefore, the survival of these patients depends on lifelong ventilatory support during sleep. Reactive oxygen species (ROS)-induced oxidative stress is a recognized risk factor involved in the pathogenesis of several chronic diseases. Therefore, monitoring systemic oxidative stress could provide important insights into CCHS outcomes. Because ROS-induced oxidative products are excreted as stable metabolites in urine, we performed an HPLC-MS/MS analysis for the quantitative determination of the three main representative oxidative biomarkers (i.e., diY, MDA, and 8-OHdG) in the urine of CCHS patients. Higher levels of urinary MDA were found in CCHS patients compared with age-matched control subjects. The noteworthy finding is the identification of urinary MDA as relevant biomarker of systemic oxidative status in CCHS patients. This study is a concise and smart communication about the impact that oxidative stress has in CCHS, and suggests the monitoring of urinary MDA levels as a useful tool for the management of these patients.

## 1. Introduction

Congenital Central Hypoventilation Syndrome (CCHS) is a rare disease of the autonomic nervous system (ORPHA:661) with an estimated incidence up to 1/200,000 live births [1,2]. Paired-like homeobox 2b (*PHOX2B* gene) is essential for neuronal formation and differentiation, especially in the autonomic nervous system, and was identified in 2003 as the CCHS-causing gene. Approximately 90% of CCHS patients are heterozygous for polyalanine repeated expansion (PolyALA) in the *PHOX2B* gene [3].

CCHS patients experience episodes of severe hypoventilation during sleep and during routine activities of daily life (i.e., studying, TV watching, computer playing, etc...), resulting from an abnormal control of breathing depending on the reduced sensitivity to hypercapnia and hypoxemia [4]. This phenomenon—known as “forgotten breathing”—implies in CCHS patients the need for lifelong ventilation support during sleep and, in more severe cases, even during wakefulness [1,2]. CCHS has no resolutive drug therapy [3,4], but early diagnosis and management of CCHS with lifelong ventilatory support have been proven to help CCHS patients live fulfilling lives.

In 2018, we provided first-line evidence of increased levels of reactive oxygen species (ROS) in erythrocyte cells and leukocyte subpopulations of CCHS patients by a cytofluorimetric analysis. No association was found between ROS levels in blood cells and other CCHS patient features such as age, gender, mutation type, and type of ventilatory support [5].

Blood cells represent an excellent model for the assessment of oxidative status; however, the blood sampling procedure is not always well-tolerated in these patients who frequently undergo medical procedures [6].

Therefore, the urinary biomarkers of oxidative stress represent a viable and appealing alternative to study oxidative status, while also taking into account the low organic and metal content of the samples which minimizes the oxidation during collection and storage [7].

**Figure 1:**
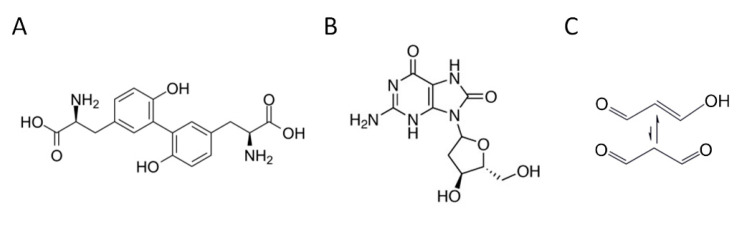
Chemical structure of the main representative oxidative stress biomarkers in urine. (**A**) o,o’-dityrosine (diY); (**B**) 8-hydroxy-2′-deoxyguanosine (8-OHdG); (**C**) malondialdehyde (MDA).

A large body of literature describes ROS as products of a controlled cellular physiological process. However, oxidative stress, resulting from an imbalance between the production of radical species and antioxidant defense systems, represents a preliminary event in the etiology of several diseases [8]. Indeed, due to their high reactivity, chronic exposure to ROS causes severe damage to most biomolecules, including lipids, proteins, and DNA [9]. These ROS oxidation products are excreted in measurable concentrations in the urine and their quantification may provide evidence of systemic oxidative status [7]. An ROS attack on proteins can result in the formation of o,o’-dityrosine (diY), a stable and reliable protein oxidation biomarker (Figure 1A). The ROS-induced oxidation of DNA may cause purine- and pyrimidines-associated lesions, among which is the hydroxylation of deoxyguanosine to form 8-hydroxy-2′-deoxyguanosine (8-OHdG), a stable end-product of non-enzymatic DNA oxidation (Figure 1B). The peroxidation of polyunsaturated fatty acids produces malondialdehyde (MDA), one of the most common lipid oxidation indicators (Figure 1C).

In CCHS, life expectancy and the extent of any intellectual disabilities depend not only on the severity of the disorder, but also on the timeliness of diagnosis and treatment. In this perspective, monitoring the subclinical state of ROS-induced systemic cellular damage in CCHS patients may represent a valuable tool for the development of early intervention strategies. For this reason, this preliminary study aims to detect urinary biomarkers of oxidative stress as a possible tool to track the progression of CCHS across the lifespan. Therefore, in this work, we used high-pressure liquid chromatography with a tandem mass spectrometry analysis (HPLC-MS/MS) to quantify key oxidative biomarkers (diY, 8-OHdG, and MDA) in the urine of CCHS patients.

## 2. Materials and Methods

### 2.1. Reagents and Chemicals

The following standards, reagents, and cartridges have been used: L,L’-dityrosine (diY), (Toronto Research Chemicals, North York, ON, Canada); o,o’-dityrosine ring ^13^C_12_, 99% (^13^C_12_-diY), (Cambridge Isotope Laboratories, Tewksbury, MA, USA); 8-hydroxy-2′-deoxyguanosine (8-OHdG), (Cambridge Isotope Laboratories, Tewksbury, MA, USA); 8-hydroxy-2′-deoxyguanosine ^15^N_5_, 98% (^15^N_5_-8-OHdG), (Cambridge Isotope Laboratories, Tewksbury, MA, USA); 1,1,3,3-Tetraethoxypropane-1,3-D_2_ (D_2_-TEP), (Cambridge Isotope Laboratories, Tewksbury, MA, USA); 1,1,3,3-tetraethoxypropane (TEP), 2,4-Dinitrophenylhydrazine (DNPH), methanol, acetonitrile and water (LC-MS grade, Biosolve), ethyl acetate, Surine^TM^ Negative Urine Control, (Merck KGaA, Darmstadt, DA, Germany); hydrochloric acid (Carlo Erba, Milan, MI, Italy); formic acid (Regis Technologies, Morton Grove, IL, USA); SPE Bond Elut NEXUS cartridges (Agilent Technologies, Santa Clara, CA, USA).

### 2.2. Study Population and Urine Sample Collection

For this study, 24 healthy controls and 12 CCHS patients with confirmed *PHOX2B* gene mutation were enrolled. The enrolled subjects were all of the same ethnicity. Healthy controls were selected from a cohort of age-matched subjects with no diseases, non-smokers, and not taking drugs or alcohol. Table 1 shows the demographic and clinical characteristics of the enrolled CCHS patients. All patients were not presented with additional features of the CCHS phenotype (i.e., neural crest tumor, Hirschsprung’s disease).

Daily activities or individual-dependent factors play an important role in ROS production [10]. Accordingly, to exclude ROS production due to acute exposure to individual factors or daily activities, CCHS patients were hospitalized the day before sample collection. In addition, to rule out any possible interference from recent hypoxic episodes, all CCHS patients underwent a complete nocturnal polysomnographic examination and capnography; their apnea-hypopnea index, oxygen desaturation index, and carbon dioxide levels were all within normal limits.

Urine samples from all enrolled subjects were collected in the morning; for CCHS patients, urine was collected after the sleep study. Samples were stored at −20 °C within 2 h of collection.

### 2.3. HPLC–MS/MS Analysis

#### 2.3.1. Calibration Standards of Urine Oxidative Biomarkers

For the determination of 8-OHdG and diY in urine, a mix solution standard of ^15^N_5_-8-hydroxy-2′-deoxyguanosine (^15^N_5_-8-OHdG) at 15 ng/mL and ^13^C_12_-o,o′-dithyrosine (^13^C_12_-diY) at 150 ng/mL in water was prepared [11].

D_2_-malondialdehyde (D_2_-MDA) was prepared at the concentration of 10 µg/mL by hydrolysis of its precursor 1,1,3,3-tetraethoxypropane-1,3-D_2_ (D_2_-TEP). An aliquot of D_2_-TEP was diluted in 0.2 N HCl and maintained at room temperature for 2 h to facilitate the total transformation of D_2_-TEP into D_2_-MDA as reported in the literature [12].

The D_2_-malondialdehyde (D_2_-MDA) obtained was diluted to a concentration of 1 µg/mL in MeOH, and was used for the quantification of MDA, extracted, and concentrated from the urine, using a solid phase extraction (SPE) cartridge.

For the determination of MDA, a 2,4-dinitrophenylhydrazine (DNPH) derivatizing agent was used to obtain MDA-DNPH as reported in the literature. DNPH is commonly used to derivatize aldehydes and ketones, to increase sensitivity and specificity in mass spectrometry [12].

#### 2.3.2. Chromatographic Separation and Selected Parameters

All samples were analyzed on an HPLC system 1100 Infinity Series Capillary Pump (Agilent Technologies) coupled to an API 4000 triple quadrupole mass spectrometer (AB SCIEX) equipped with Electrospray (ESI) operating in positive ion mode and Multiple Reaction Monitoring (MRM) mode.

Chromatographic separations were performed using the chromatographic column Eclipse plus C18, 3.5 µm, 4.6 *×* 100 mm (Agilent Technologies), located in the oven at a temperature of 40 °C, and the total run time analysis was 21 min. Gradient elution was performed with mobile phase A (water + 0.1% formic acid) and mobile phase B (MeOH/ACN (70/30 *v/v*) + 0.1% formic acid) as follows: 5% B maintained for 2 min, to 50% B in 0.1 min., to 60% B from 2.1 to 15 min, then back to 5% B in 0.1 min and re-equilibration for 8 min. The mobile phase was set at a flow rate of 0.4 mL/min.

The capillary voltage was set to 5000 V, and heated turbo gas (air) with a flow rate of 10 L/min was used at 400 °C. Transitions monitored in MRM mode are summarized in Table 2.

Data acquisitions were performed by Analyst software version 1.5.2. (AB SCIEX) which includes the “Explore” option (for chromatographic and spectral interpretation) and the “Quantitate” option (for quantitative information generation) constructed with the Quantitation Wizard.

#### 2.3.3. HPLC-MS/MS Analysis of Urine Biomarkers

Two analyses were chosen to determine diY, 8-OHdG, and MDA urine biomarkers in the matrix. For diY and 8-OHdG, 100 µL aliquot of urine was added to 200 µL of water containing a mixture of labeled standards ^13^C_12_-o,o′-dithyrosine (150 ng/mL) and ^15^N_5_-8-hydroxy-2′-deoxyguanosine (15 ng/mL), and finally a volume of 3 µL was injected into HPLC-ESI-MS/MS.

MDA was quantified after derivatization with 2,4-dinitrophenylhydrazine (DNPH). A 20 µL of labeled standard D_2_-MDA (1 µg/mL) was added to an aliquot of 500 µL of the same urine sample and 200 µL of DNPH 0.005 M.

The samples were kept at room temperature for 30 min and then an SPE was performed with Bond Elut Nexus Cartridges.

Recovery from SPE was obtained with 1 mL of MeOH and 1 mL of ethyl acetate, evaporated under nitrogen at 50 °C, then reconstituted and diluted with water/methanol (80:20 *v/v*). A volume of 10 µL was injected into HPLC-ESI-MS/MS.

Chromatographic separations were performed according to parameters reported above and available in Figures S1–S3 in Supplementary Materials.

The quantitation results (as the mean of the triplicate sample analysis) were based on the isotopic dilution, expressed in µmol/mol creatinine, to reduce both intra- and inter-individual variability.

### 2.4. Statistics

For the statistical analysis of oxidation biomarkers, all subjects were divided into two age groups: minors (<18 years: age 3–15 years old), and adults (>18 years: age 19–32 years old).

The differences between sample groups were statistically evaluated using the unpaired *t*-test.

## 3. Results and Discussion

Oxidative Stress Urine Biomarkers in CCHS Patients

Levels of diY, 8-OHdG, and MDA were assessed as stable oxidative products representative of oxidative damage to protein, DNA, and polyunsaturated fatty acids, respectively, by an HPLC-MS/MS analysis of urine samples.

In this work, all enrolled subjects were divided into two age groups, namely minors (<18 years: age 3–15 years old), and adults (>18 years: age 19–32 years old).

**Figure 2:**
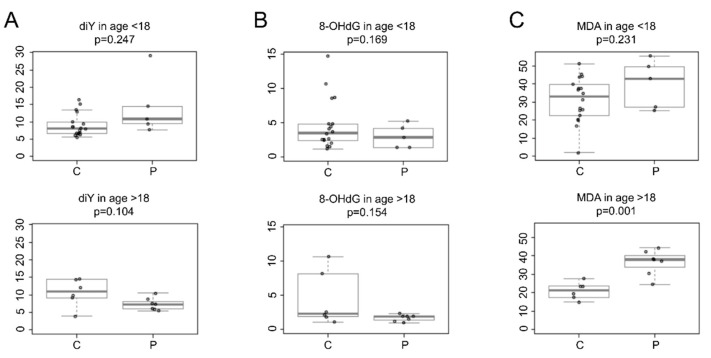
Levels of urinary oxidative biomarkers in healthy controls (**C**) and CCHS patients (P) from two different age groups, i.e., <18 years and >18 years. Boxplots represent the concentration levels (μmol/mol creatinine) of (**A**) o,o′-dityrosine (diY), (**B**) 8-hydroxy-2′-deoxyguanosine (8-OHdG), and (C) malondialdehyde (MDA). Values were corrected with urinary creatinine content. *p*-values were obtained with a two-class *t*-test.

Figure 2 shows the oxidative biomarkers quantified in the urine of CCHS patients and age-matched healthy controls.

Specifically, CCHS patients of both age groups showed no significant difference in urinary diY levels compared to healthy control subjects (minors: patients mean 14.3, SD 8.6, *n* = 5 vs. controls mean 9.0, SD 3.2, *n* = 18, *p* = 0.247); (adults: patients mean 7.3, SD 1.7, *n* = 7 vs. controls mean 10.6, SD 3.9, *n* = 6, *p* = 0.104), (Figure 2A).

Similarly, urinary 8-OHdG levels were not statistically different in patients compared to healthy controls of both age groups (minors: patients mean 3.0, SD 1.7, *n* = 5 vs. controls mean 4.7, SD 3.7, *n* = 18, *p* = 0.169); (adults: patients mean 1.7, SD 0.47, *n* = 7 vs. controls mean 4.4, SD 3.99, *n* = 6, *p* = 0.154), (Figure 2B).

Urinary MDA levels were comparable between CCHS patients aged <18 years and healthy control subjects (patients mean 39.9, SD 13.39, *n* = 5 vs. controls mean 31.1, SD 12.34, *n* = 18, *p* = 0.231). There was a wide variability in urinary MDA levels in minors (aged <18 years). This variability could be explained by the different stages of physical development of the enrolled pediatric subjects (3–15 years), and the small number of samples from CCHS minors. Recall that CCHS is a rare genetic disease that affects few individuals among live births. In addition, sample collection from CCHS minors was restricted because of study limiting factors, i.e., the difficulty in collecting urine from CCHS minors (aged <3 years) and the wide distribution of patients in the Italian territory, which made it difficult to reach the CCHS Center (Meyer Children’s Hospital in Florence, Italy). However, this study shows that the mean urinary MDA levels were significantly different among CCHS patients aged >18 years compared to healthy control subjects (patients mean 36.2, SD 6.8, *n* = 7 vs. controls mean 21.0, SD 4.6, *n* = 6, *p* = 0.001), (Figure 2C).

Since there were no patients presenting hypoventilation while also awake, the apnea-hypopnea index, the oxygen desaturation index, and carbon dioxide levels of CCHS enrolled patients were all within normal limits during nocturnal ventilation, and we can exclude an association between these results and the eventual ineffectiveness of artificial ventilation.

High lipid peroxidation has already been correlated with chronic diseases (e.g., diabetes mellitus, atherosclerosis, and inflammation) [13]. Due to its relative stability, urinary MDA is of particular interest as a biomarker of oxidative stress to evaluate lipid peroxidation. Accordingly, MDA represents a key metabolite for assessing the extent of biological membrane damage as a primary consequence of oxidative stress in patients with CCHS.

This noteworthy finding reflects a higher oxidative status in CCHS patients; these data are particularly evident in adult CCHS, albeit of a younger age (age 19–32 years).

The difference in MDA levels may be due to long-term oxidative stress that causes progressively greater oxidative damage with advancing age that may contribute to the development of chronic oxidative-related disorders. Scientific evidence reports that the aging process is linked to increased oxidative damage, and that oxidative stress is involved in several age-related conditions [9]. Accordingly, it is important to monitor the aging process of CCHS patients from a young age. This could allow early interventions to contain the further increase of ROS in aging and the related onset of other chronic diseases. Indeed, it is important to look at CCHS in its complexity, in addition to the respiratory disorder and the mandatory need for ventilatory support.

As CCHS has no drug therapy, this study highlights the importance that the evaluation of the urinary MDA levels may have in identifying an individual biochemical shift towards chronic oxidative-related diseases, especially in the early prodromal phase. This is especially important given that CCHS patients have been shown to have widespread and progressive brain damage [14]. Furthermore, curcumin, a well-known antioxidant agent, has been shown in in-vitro experiments to promote the cellular clearance of toxic aggregates containing *PHOX2B* mutated proteins [15].

To date, it remains unclear whether increased oxidative stress and its subsequent damage are pathophysiological conditions of CCHS or whether *PHOX2B* genotypic factors are involved. However, our findings suggest that early detection of oxidative damage in CCHS patients might allow a more individualized and sensitive profile of the risk of worsening their pathophysiological conditions in the long term. Lifespan clinical monitoring of risk factors in CCHS patients could have prognostic value in addition to conventional clinical monitoring.

## 4. Conclusions

This concise communication serves as a smart disclosure to the CCHS scientific community that CCHS is associated with a higher systemic oxidative status, and that urinary MDA represents the key biomarker of oxidation. Knowing the level of impairment of systemic oxidative status could help stratify the risk in CCHS patients.

In summary, these results are hypothesis-generating for future clinical studies aimed at improving the outcomes of CCHS by hindering increased oxidative stress, through the promotion of a heathy lifestyle, a correct diet, and the therapeutic use of antioxidant agents.

## Figures and Tables

**Table 1:** Demographic and clinical characteristics of enrolled CCHS patients.

ID	Group	Age	Sex	PHOX-2B Gene Mutation	Type of Ventilation
1 2		9	F	PolyALA 20/26	Tracheostomy
		14	F	PolyALA 20/26	Tracheostomy
3 4 5 6	<18 years	3	M	PolyALA 20/26	Non-Invasive Ventilation
		15	F	Frameshift	Tracheostomy
		3	F	Frameshift	Tracheostomy
		19	F	PolyALA 20/25	Pacemaker/Non-Invasive Ventilation
7 8		25	F	PolyALA 20/25	Non-Invasive Ventilation
		23	F	Frameshift	Non-Invasive Ventilation
9	>18 years	24	F	PolyALA 20/26	Non-Invasive Ventilation
10		32	M	PolyALA 20/26	Non-Invasive Ventilation
11		21	M	PolyALA 20/26	Non-Invasive Ventilation
12		19	F	PolyALA 20/27	Non-Invasive Ventilation

**Table 2:** MRM mode transitions used for the three urine biomarkers.

MRM Unlabeled Standard	MRM Labelled Standard
diY 361.3 > 315.3	^13^C_12_-diY 373.3 > 327.3
8-OHdG 284.3 > 168.3	^15^N_5_-8-OHdG 289.3 > 173.3
MDA-DNPH 235.3 > 159.3	D_2_-MDA-DNPH 237.3 > 161.3

## Data Availability

Not applicable.

## Supplementary Materials

The following supporting information can be downloaded at: https://www.mdpi.com/article/10.3390/antiox11050929/s1, Figure S1: Chromatographic separation of diY; Figure S2: Chromatographic separation of 8-OHdG; Figure S3: Chromatographic separation of MDA-DNPH.
