# Supplementary material for: Urinary Biomarkers as a Proxy for Congenital Central Hypoventilation Syndrome Patient Follow-Up
Source: Antioxidants (Basel). 2022 May 9;11(5):929. doi: 10.3390/antiox11050929 (PMC9138029; doi:10.3390/antiox11050929)
Supplement: Supplementary file 1 [file antioxidants-11-00929-s001.zip › antioxidants-1634619-supplementary.pdf]

Supplementary Materials

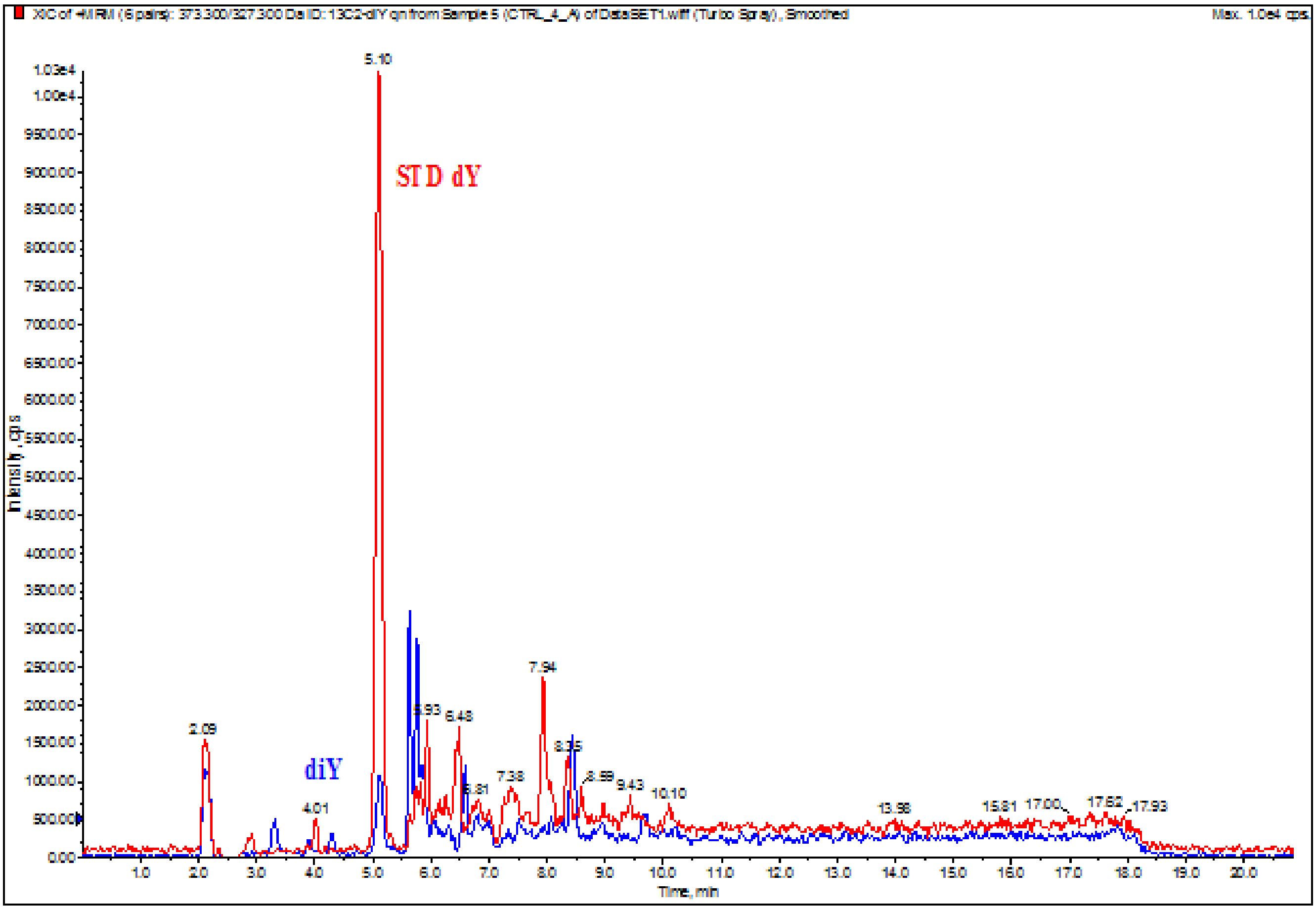

Figure S1. Chromatographic separation of diY, tR = 5.1 min.

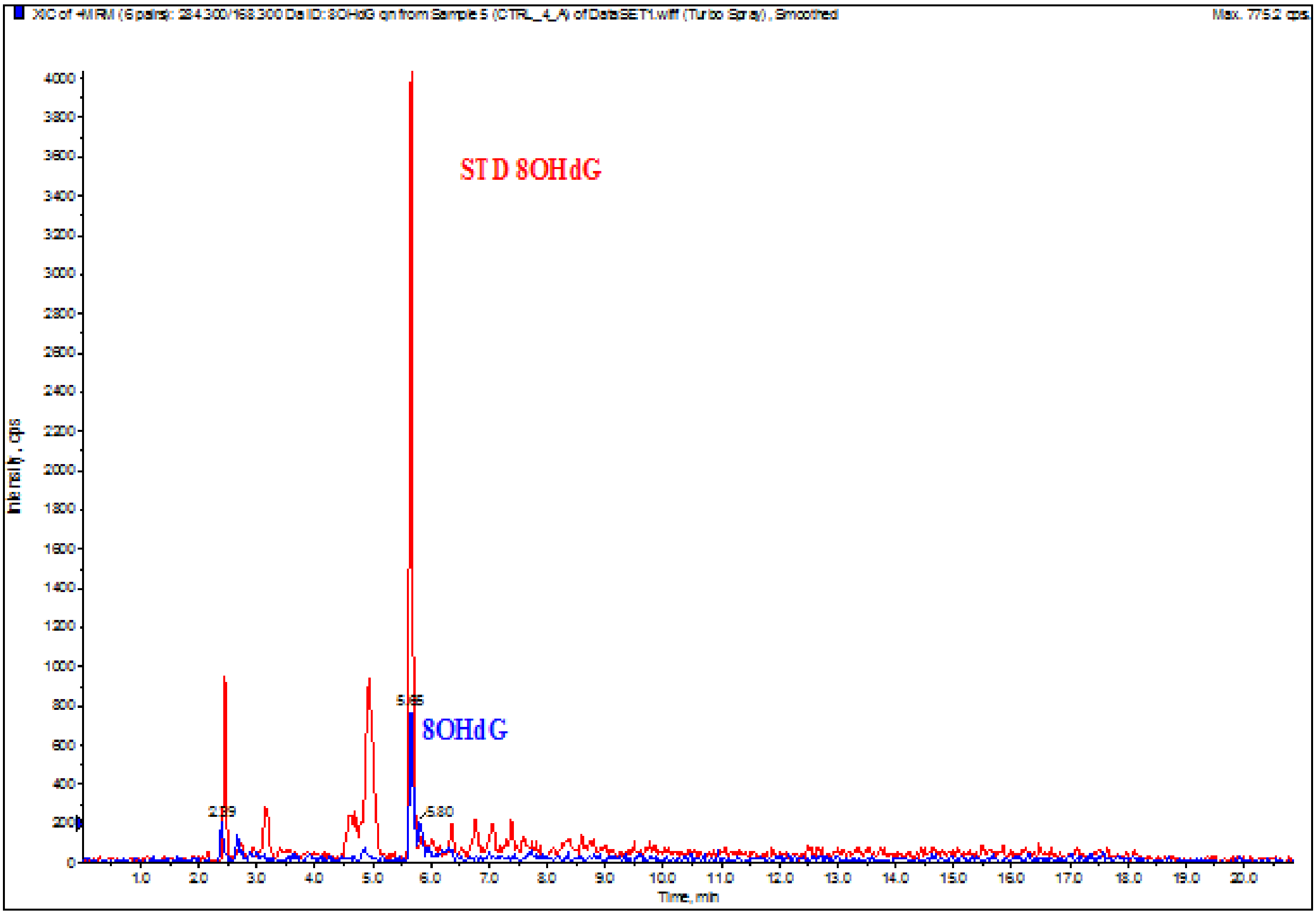

Figure S2. Chromatographic separation of 8-OHdG, tR = 5.6 min.

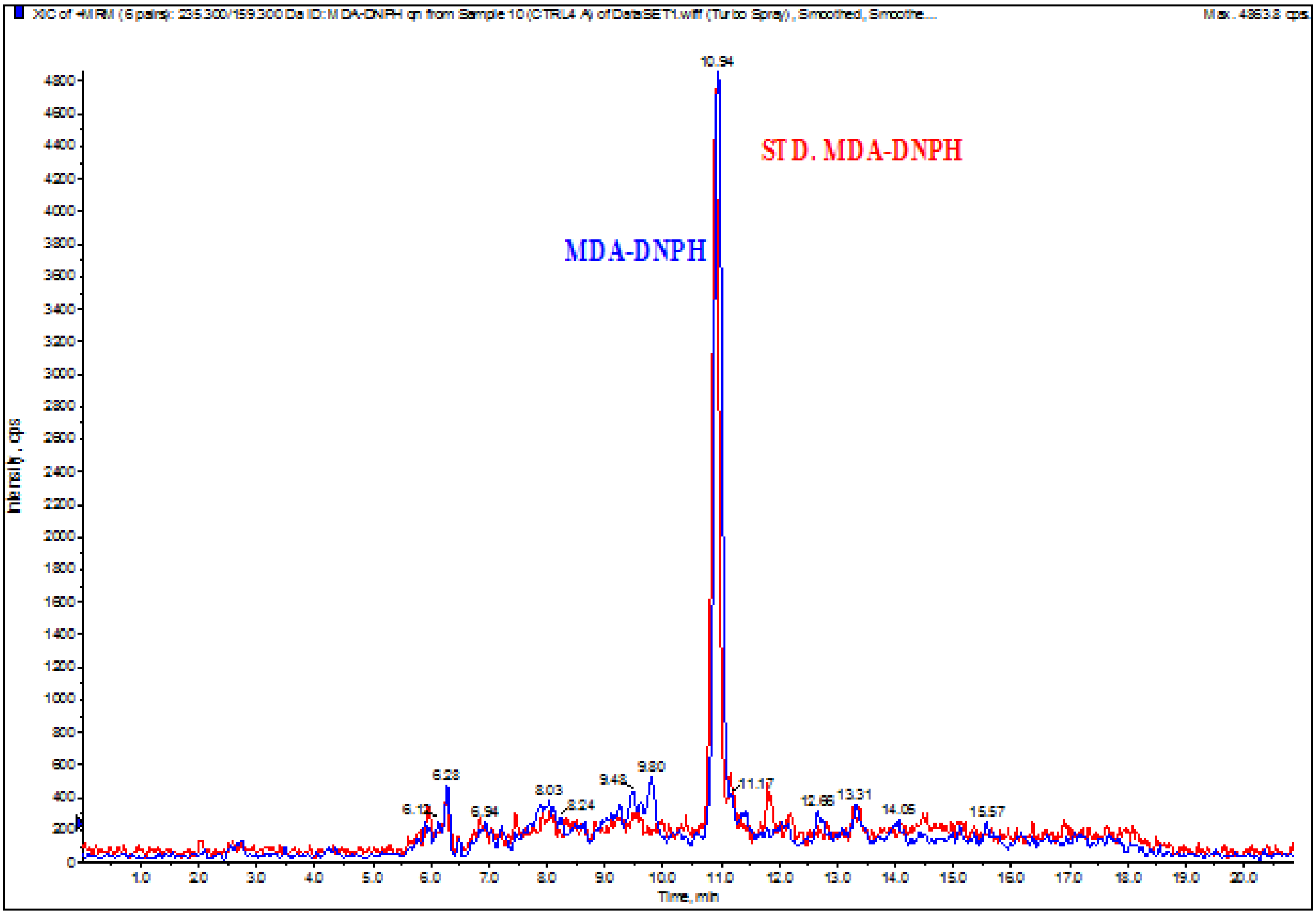

Figure S3. Chromatographic separation of MDA-DNPH, t_R = 10.9 min.
